# Training and onboarding initiatives in high energy physics experiments

**DOI:** 10.3389/fdata.2025.1497622

**Published:** 2025-02-10

**Authors:** Allison Reinsvold Hall, Nicole Skidmore, Gabriele Benelli, Ben Carlson, Claire David, Jonathan Davies, Wouter Deconinck, David DeMuth, Peter Elmer, Rocky Bala Garg, Stephan Hageböck, Killian Lieret, Valeriia Lukashenko, Sudhir Malik, Andy Morris, Heidi Schellman, Graeme A. Stewart, Jason Veatch, Michel Hernandez Villanueva

**Affiliations:** ^1^Physics Department, United States Naval Academy, Annapolis, MD, United States; ^2^Department of Physics, University of Warwick, Coventry, United Kingdom; ^3^Department of Physics, Brown University, Providence, RI, United States; ^4^Physics Department, Westmont College, Santa Barbara, CA, United States; ^5^Physics and Astronomy, University of Pittsburgh, Pittsburgh, PA, United States; ^6^African Institute for Mathematical Sciences, Cape Town, South Africa; ^7^Department of Physics and Astronomy, University of Manchester, Manchester, United Kingdom; ^8^Physics and Astronomy, University of Manitoba, Winnipeg, MB, Canada; ^9^Department of Science, Valley City State University, Valley City, ND, United States; ^10^Department of Physics, Princeton University, Princeton, NJ, United States; ^11^Physics Department, Stanford University, Stanford, CA, United States; ^12^European Organization for Nuclear Research (CERN), Geneva, Switzerland; ^13^Physics Institute, University of Zurich, Zurich, Switzerland; ^14^Institute for Nuclear Research, National Academy of Sciences of Ukraine, Kyiv, Ukraine; ^15^Physics Department, University of Puerto Rico Mayaguez, Mayaguez, Puerto Rico; ^16^CNRS/IN2P3, CPPM, Aix Marseille University, Marseille, France; ^17^Department of Physics, Oregon State University, Corvallis, OR, United States; ^18^Department of Physics and Physical Sciences, California State University Stanislaus, Turlock, CA, United States; ^19^Physics Department, Brookhaven National Laboratory, Upton, NY, United States

**Keywords:** training, onboarding, analysis software, scientific computing, data analysis, high energy physics, particle physics

## Abstract

In this article we document the current analysis software training and onboarding activities in several High Energy Physics (HEP) experiments: ATLAS, CMS, LHCb, Belle II and DUNE. Fast and efficient onboarding of new collaboration members is increasingly important for HEP experiments. With rapidly increasing data volumes and larger collaborations the analyses and consequently, the related software, become ever more complex. This necessitates structured onboarding and training. Recognizing this, a meeting series was held by the HEP Software Foundation (HSF) in 2022 for experiments to showcase their initiatives. Here we document and analyze these in an attempt to determine a set of key considerations for future HEP experiments.

## 1 Introduction

Onboarding refers to the process of integrating new members into an organization and providing them with the knowledge and skills to become effective members of said organization. The software used in High Energy Physics (HEP) experiments is becoming increasingly complex in order to handle the unprecedented data volumes experiments can now record; as a consequence it has become more challenging for new members to gain proficiency with. Moreover, with collaborations often consisting of more than 1000 members spanning multiple continents, induction into the collaborations' day-to-day activities and inner structure is an equally important component of the onboarding process. It is therefore important that experiments have effective and sustainable software training and induction programs. Whilst software frameworks are experiment-specific, there is great value in comparing onboarding and training strategies across different HEP collaborations. Challenges such as “How do we run effective training events for collaboration members spread across multiple timezones?” and “How do we encourage current collaboration members to provide this training to newcomers?” are common. We also describe how the COVID-19 pandemic changed the training paradigm for the experiments.

The consensus within the HEP community on the need for software training programs for researchers more generally is reflected in the HEP Software Foundation (HSF)^1^ Community White Paper (Albrecht et al., 2019; HEP Software Foundation et al., 2019). DIANA-HEP^2^, IRIS-HEP^3^ and the HSF organized workshops bringing together the training experiences and needs of different experiments to produce a common software curriculum. Following the initial workshop^4^ by the HSF in 2020, a meeting series^5^ was held by the HSF Data Analysis Working Group in 2022 for experiments to showcase both their software training and collaboration induction initiatives. This paper summarizes their approaches and discusses key points for cross-experiment consideration.

In this paper, Section 2 describes how training and induction activities are organized in individual experiments. Section 1 analyzes these initiatives and discusses the general themes, and Section 3.2 outlines key considerations for future experiments when creating training and induction events and materials.

## 2 Experiment approaches

The meeting series hosted by the HSF Data Analysis Working Group brought together several experiments to present and discuss their software training and induction initiatives for new collaboration members. Each experiment's approach is described below.

### 2.1 ATLAS

**Members: 6000+, Institutes: 182, Countries: 42**

A Toroidal LHC ApparatuS, nicknamed the ATLAS experiment (ATLAS Collaboration et al., 2008), is situated at point 1 of CERN's Large Hadron Collider (LHC) (Evans and Bryant, 2008). ATLAS is a general-purpose detector dedicated to investigating a diverse array of physics topics. This includes precision measurements within the Standard Model (SM) with a prime focus on the properties of the Higgs boson and searches for Beyond-the-Standard-Model (BSM) physics. ATLAS boasts an exceptionally active and devoted physics program dedicated to both proton physics and heavy-ion physics. With over 6000 members and 3000 scientific authors, hailing from 182 institutions across 42 countries, ATLAS represents one of the most ambitious and cooperative experiments ever realized in the history of scientific research^6^.

The ATLAS collaboration has been organizing specialized training for analysis software and workflows since 2004. However, these events became more comprehensive and regularized with the start of the LHC in 2008. The training programs are carefully designed to cater to both new members of the collaboration and experienced individuals seeking deeper insights. Up until 2020, these training events were conducted in-person at CERN, without any remote connection. However, the outbreak of the COVID-19 pandemic prompted a paradigm shift, leading ATLAS to transform their software tutorials from in-person to remote sessions, featuring pre-recorded lectures and interactive question-answer sessions. This approach enabled participants from all corners of the globe to access the training resources remotely and therefore ATLAS decided to continue the remote sessions. Today, ATLAS employs a variety of different tutorials to effectively educate its members, as detailed below (ATLAS Collaboration, 2024).

#### 2.1.1 Primary induction to collaboration and core software training

**ATLAS induction day and analysis software tutorial**

ATLAS runs a comprehensive week-long tutorial comprising two main segments: the first day, known as the induction day, serves to introduce participants to the ATLAS experiment and provides a broader perspective of working in a HEP research environment. A part of the induction day is reserved to guide students through the process of setting up their CERN and ATLAS computing accounts, making this complex process easier for them.

The induction day is organized by the ATLAS Early Career Scientist board,^7^ this event caters to the students who have already completed their first year in the ATLAS collaboration and have likely finished their qualification projects leading to ATLAS authorship. During this half-day event, participants learn about the finer intricacies of the ATLAS organization and its mentoring framework. The event also sheds light on specific processes, such as accumulating Operation Task Points (OTPs) and securing speaking opportunities at national and international conferences, through detailed presentations. A question-answer session is held at the end to provide participants with the chance to seek clarifications, gain deeper insights, and engage directly with the experts.

The subsequent four days are wholly devoted to data analysis software training: the ATLAS Analysis Software Tutorial. In a recent development, the software tutorial underwent a structural transformation aimed at enhancing its pedagogical value and adopting a project-based approach where participants are guided through the process of conducting an end-to-end analysis. The current implementation of this new framework involves the application of the 2^nd^ generation LeptoQuark (LQ) analysis on ATLAS Run 2 data (ATLAS Collaboration et al., 2020). Throughout the tutorial week, students are expertly guided through each step of the analysis using a combination of presentations and interactive hands-on-tutorials. Wherever possible, code is provided in the form of Jupyter notebooks, streamlining the processes of code editing and execution. Students are encouraged to work together to promote team-building and networking. The first trial of this new format was performed in September 2022 at SLAC^8^, yielding remarkable success and great feedback from the students. Currently, this tutorial is organized quarterly at CERN, offering back-to-back sessions for in-person and remote participation and annually in the USA for in-person participants.

#### 2.1.2 Further software training beyond core curriculum

**ATLAS Software Development tutorial**

The ATLAS Software Development tutorial is designed to help train those who will pursue technical computing projects. These dedicated training sessions assume that the participants have already followed the software induction tutorial and possess a solid foundation in programming languages such as C++, Python, and the ATLAS software Athena (ATLAS Collaboration, 2019). The core objective of this tutorial is to enhance participants' proficiency in writing high-quality, error-free, and sustainable code. The curriculum includes essential yet complex subjects like multithreading, databases, Git version control, code debugging, and more. Through a blend of presentations and hands-on exercises, participants are equipped with practical skills and theoretical knowledge, fostering a comprehensive learning experience.

#### 2.1.3 Sources of continued training and support

ATLAS maintains well-structured software documentation,^9^ which includes user-friendly guides for essential tools like Git and CMake, along with a comprehensive guide for the ATLAS software Athena (ATLAS Collaboration, 2019). Moreover, to facilitate learning at one's own pace, ATLAS provides a self-guided software tutorial^10^ that remains readily accessible to all when required. Alongside this, ATLAS regularly organizes various workshops targeting specific topics such as detector upgrades, tracking mechanisms, flavor-tagging tools etc. that are open to all collaboration members.

There are also numerous active mailing lists and e-groups available that allow individuals to quickly connect with experts for technical support.

#### 2.1.4 Other learning resources

ATLAS is actively engaged with various social media platforms, utilizing them as avenues to disseminate knowledge. Notably, ATLAS shares educational content through its YouTube channel^11^ where a wealth of videos provide in-depth insights into the ATLAS detector and its underlying physics. This resource not only proves invaluable for new collaboration members seeking knowledge but also serves as a means for active members to deepen their knowledge.

### 2.2 CMS

**Members: 6000+, Institutes: 240, Countries: 50**

The Compact Muon Solenoid (CMS) experiment (CMS Collaboration, 2008) at the CERN LHC is an all-purpose detector that is used to perform precision measurements of the SM, test properties of the Higgs boson, and look for evidence of BSM physics such as dark matter or supersymmetry. The CMS Collaboration has over 6000 members, including engineers, undergraduate students, and approximately 2100 Ph.D. physicists and 1200 doctoral students^12^.

CMS offers several different training events to introduce new members to the collaboration and teach essential analysis and software development skills. Many of these events emphasize hands-on practice and networking opportunities.

#### 2.2.1 Primary induction to collaboration and core software training

**CMS induction course**

To introduce newcomers to the collaboration, CMS offers an Induction Course once or twice a year. This course focuses on opportunities to learn the collaboration structure and gain an overview of the experiment, rather than teaching hands-on programming skills. The course is split into two days and is currently held in hybrid mode, with both in-person and remote options, and is fully recorded. Lectures generally include talks from the experiment spokesperson and physics coordination; overviews of the different aspects of the experiment including detector subsystems, trigger, offline and computing; and an introduction to groups such as the Diversity Office, Communications Office, and the CMS Secretariat. So far, the course has been offered 15 times since 2014.

**CMS data analysis school**

To learn analysis software, the most complete training offered is the CMS Data Analysis School^13^ (DAS). This event was started in 2011 and has been offered 2-3 times per year since then. DAS takes place over an entire week, usually at either the LHC Physics Center (LPC) at Fermilab or at CERN, although other locations (Pisa, Taipei, DESY, Kolkata, Bari, Daegu, Beijing) have also hosted. The school is geared toward new Ph.D. students, but is also attended by some undergraduate students as well as by postdocs or junior faculty without previous CMS analysis experience. The typical attendance is 50–70 students.

The DAS structure emphasizes hands-on exercises and teamwork. Over the years the format has evolved slightly, but the basic structure is the following:

- **Mandatory pre-exercises** are used to get all students set up with the proper computing accounts and familiar with the basics so they are able to hit the ground running once they arrive. The deadline for completion is before the start of the school.
- **Lectures** on the first two days introduce students to key concepts about analysis as well as the collaboration in general. Topics include: CMS Physics, Introduction to the LHC and the CMS Detector, Software/Analysis Tools, Diversity and Inclusion, and Communications/Outreach.
- **Short exercises** are two-hour sessions that cover the essentials of specific objects and basic analysis ingredients, such as muons, jets, tracking, statistics, etc.
- **Writers PUB** is led by senior CMS members, who explain the publication process of a CMS paper.
- **Long exercises** are the core of the DAS experience. The students are divided into small analysis teams and, with guidance from facilitators, seek to finish a complete physics analysis by the end of the week.
- A **mini-symposium** on the last day wraps up the school. All teams present their results from the long exercise, and a panel of judges decide on the best presentation. Members of the winning team typically receive a small prize (like an LPC coffee mug) as well as coveted bragging rights.

Networking is an important component throughout the school. Each participant has a commitment and responsibility to their long exercise analysis team, which is the key to their individual and team success. While all team members participate in the same long exercise, team members are assigned different short exercises so that the team as a whole can have full coverage of the needed tools for the long exercise analysis. All team members participate in preparing the slides, and all team members are required to present during the final mini-symposium at the end. The DAS participants are also supported throughout via channels setup on the open-source, online chat platform Mattermost^14^.

DAS is viewed as an essential data analysis “boot camp”, but its success requires a major effort from many people. There are typically close to 50 facilitators per DAS, including many postdocs who are responsible for leading the short and long exercises. At the LPC, many of the facilitators are part of the LPC Distinguished Researcher program^15^, which provides funding for postdocs and scientists/faculty to stay at Fermilab and contribute to the LPC community by leading training events, physics analyses, and hardware or software efforts. Many postdocs and senior graduate students once participated in DAS as students and now choose to serve as facilitators. Two full-time LPC support staff members, the two LPC co-coordinators (who serve two-year appointments), and the CMS Schools Committee guide the overall organization.

To adapt to the COVID-19 pandemic, the LPC hosted a virtual DAS in January 2021 and 2022. Instead of one week, the event was spread out over two weeks to mitigate the fatigue from spending too many hours videoconferencing per day. The virtual DAS was still successful in teaching core analysis skills, but it was harder to maintain the crucial networking aspect of the school. Fortunately, in January 2023 the LPC was able to once again host DAS in-person.

#### 2.2.2 Further software training beyond core curriculum

**Hands-on tutorial sessions**

For training on specific topics, the Fermilab LPC offers Hands-On Tutorial Sessions^16^ (HATS) during the spring and summer each year. There are typically about 15–20 HATS per year focused on specific physics objects or tools for data analysis such as Git, machine learning, the CMS trigger system, jet algorithms, or scientific Python. Each tutorial includes both lectures and hands-on exercises and typically takes place over one or two afternoons. Participants are encouraged to attend in-person at Fermilab, but remote participation via Zoom^17^ and Mattermost discussions are also possible. The tutorials are all recorded, with the videos and transcripts posted to the CERN Document Server (CDS) to serve as useful reference materials. Recently, there have been efforts to move the HATS to a software carpentry model (Wilson, 2006, 2016) to simplify maintenance.

**Workshops and hackathons**

Additional workshops or schools are also offered as necessary to provide training on specific topics. In recent years, these have included CMS Upgrade Schools (Filippis et al., 2017; Malik et al., 2014) to train new contributors to the HL-LHC upgrades and dedicated workshops or hackathons led by Physics Object Groups (POGs) or Detector Performance Groups (DPGs) to prepare for data taking or after the release of new tools, etc. A general CMS Physics Object School (CMSPOS) has been offered three times. CMSPOS adopted a similar structure to DAS, but with a focus on learning about physics objects such as electrons or muons in more detail. The goal of CMSPOS is to train people who will become developers for the software of particular POGs or DPGs.

#### 2.2.3 Sources of continued training and support

The LPC at Fermilab hosts several Mattermost channels and discussion forums open to all CMS members for daily communication with users for computing and analysis related issues and support.

#### 2.2.4 Other learning resources

The LPC regularly offers graduate-level advanced courses, which are organized in association with US universities so students have the option to get course credit. These courses include hybrid (in-person or remote) lectures with homework and exams and have covered a wide range of analysis-related topics, including computational physics, statistics, detectors, and machine learning.

Weekly Physics Analysis Discussions hosted remotely and in-person by the LPC provide an informal way for analyzers to ask for help and hear about the latest updates and recommendations for CMS analyses.

### 2.3 LHCb

**Members: 1600+, Institutes: 96, Countries: 21**

The LHCb detector (LHCb Collaboration, 2008) is a single-arm forward spectrometer located at point 8 of the LHC. It has a broad physics program covering *CP*-violation, rare decays, electroweak physics, and more, with often world-leading results. Currently, the collaboration consists of approximately 1600 members from 96 institutes in 21 countries. Since 2020, this represents an increase of approximately 100 new members. In terms of scientists, 28% of the members are Ph.D. students, 16% are post-doctoral researchers, and a further 27% are at a senior level. These ratios are different for technical members.

The approach to training and onboarding at LHCb is community-driven by volunteers, typically Ph.D. students, with limited oversight from academics. The volunteers organize a section of the onboarding process (typically workshops or talks, detailed below), before finding the next set of volunteers at the end of their tenure. This highly collaborative peer-to-peer system comes with several marked advantages. Firstly, fresh eyes applied to the training materials each year helps in keeping them up-to-date and relevant. Secondly, the needs of newcomers are well-known to Ph.D. students, who are in a good position to judge the appropriate level of prior knowledge that may be assumed. Thirdly, due to these events being held in person wherever possible, they also serve a social function, allowing students to meet and network with other LHCb members, improving the cohesion of early-career scientists within the collaboration. Finally, the prospect of asking questions is far less daunting when posed to more senior peers than to well-established figures within the collaboration.

#### 2.3.1 Primary induction to collaboration and core software training

**StarterKit**

Starterkit (Puig and on behalf of the LHCb Starterkit Team, 2017) is an annual introductory hands-on workshop for LHCb newcomers. The workshop was established in 2015. It was held exclusively in person until 2020, online in 2020-2021, and in hybrid mode in 2022 and belatedly in February 2024. Approximately 40 students participate in the in-person training at CERN each year, and about 100 participate online. The Starterkit is usually organized and taught by Ph.D. students and postdocs. Before the workshop, teaching tips and tricks are shared amongst volunteers based on experience from the previous year. Lectures are maintained by the LHCb community, and teachers are required to review and update lecture material prior to their lessons. The Starterkit is often the first collaboration event for participants; therefore, the spokesperson, physics coordinator, and early career, gender, and diversity officers give a short introduction to their roles within LHCb, and the work of the collaboration as whole.

The week-long Starterkit program consists of two parts and assumes no prior knowledge. The first part follows HSF analysis essentials on basic programming skills,^18^ and the second part covers the “First Analysis Steps”, which introduces LHCb-specific software.^19^ HSF analysis essentials includes hands-on lessons on Bash, Python (including selected PyHEP,^20^ packages) GitLab, and Snakemake (Burr et al., 2024). The “First Analysis Steps” section describes the LHCb dataflow, distributed analysis software, and LHCb-specific reconstruction software, as well as how to best find help at LHCb in a logical order. The last day is dedicated to a practice session, where participants use their newly acquired skills to process a subset of the LHCb dataset, just as would be done in future analysis work. In 2017-2019, the first part of Starterkit was shared with the ALICE and SHiP collaborations (HEP Software Foundation et al., 2019).

Materials from the Starterkit lectures are available all year long for self-study on a public website and serve as a useful starting point for those looking to implement unfamiliar techniques. This is also handy for those external to LHCb who are interested in LHCb Open Datasets, as the Starterkit lessons provide easy-to-follow data processing instructions. Lectures are also recorded and uploaded to the CDS. To adjust the material each year, prior to the workshop, participants are asked to fill in a quiz, which helps teachers to understand their level. After the workshop, participants provide feedback on the teaching and organization, which is used to revise the workshop further. The power of Starterkit is its collaborative spirit, peer-to-peer model, clear explanations of collaboration jargon, and annual reviews.

Starting in 2021, training in LHCb started to slowly migrate toward Run 3 software. As of December 2022, the annual Starterkit covers topics from both Run 2 and Run 3 software. In March 2022, a special online Run 3 Starterkit was organized for everyone in LHCb, with around 200 people in attendance. Each of the topics was introduced by experts, maintaining its hands-on spirit. This event formed the basis of the Run 3-related materials to be used in the Starterkit going forward. When the transition period between Run 2 and Run 3 is complete, Starterkit will only cover Run 3 topics; the Run 1 and Run 2 materials will be retired from the workshop, but the materials and lecture recordings will be kept online for self-guidance. To help coordinate the effort toward developing a standalone Run 3 StarterKit, in 2024, a new work package was set up within the LHCb Data Processing and Analysis (DPA) project. With the remit of coordinating the production of the required course content and overseeing the delivery of the StarterKit events, this workpackage facilitates timely delivery of Run 3 StarterKit and quality training events for years to come.

#### 2.3.2 Further software training beyond core curriculum

**Impactkit**

In spring 2016, a follow-on event from the Starterkit, called the Impactkit, was organized with the aim of building upon the skills and techniques learned in the Starterkit and introducing more specialized topics. Subsequent Impactkits have been held in one form or another every year since and cover topics such as simulation with Gauss,^21^ the software trigger with Moore,^22^ particle combining and filtering, and more sophisticated techniques for distributed analysis. In a similar manner to the Starterkit, the last day is typically reserved for a hackathon-style practical session, where participants are able to consolidate what they've learned by working on more open-ended problems. Slightly more compact than the Starterkit, the Impactkit typically takes place over three days, and attending both events enables participants to know all the software that is regularly used by a significant number of LHCb analysts. The documentation can be found in the same location as the Starterkit materials, under the heading of “Second Analysis Steps”.^23^

#### 2.3.3 Sources of continued training and support

In addition to formal training initiatives, the LHCb collaboration benefits greatly from the use of Mattermost. The platform supports the creation of numerous separate channels to permit discussion of specific topics. As well as facilitating easy communication within small research teams, Mattermost is a crucial resource for those seeking out timely technical support for software problems that may arise. This is particularly important for new members of the collaboration who can get their questions answered quickly by an expert by posting in the appropriate channel. While more-experienced users are under no obligation to respond to such messages, the strong community spirit of the collaboration sustains this form of troubleshooting.

#### 2.3.4 Other learning resources

**StarterTalk and theory talks**

StarterTalk is an hour-long lecture on LHCb topics organized by postdocs and held during LHCb collaboration weeks. Established in 2017, StarterTalk invites LHCb experts on LHCb detector, reconstruction, and physics topics to share their expertise with their colleagues in a pedagogical way. This helps to improve members' general understanding of the wide range of activities that the collaboration performs. Around 20 lectures had taken place by December 2022, with an average in-person attendance of 50 people. All lectures are recorded and uploaded to the CDS.

As an extension to the StarterTalks, in 2022, the first series of theory talks were organized ahead of the annual Implications Workshop^24^ that brings together theorists and LHCb members, helping each side to understand the other's needs better. These talks aimed to improve understanding within the collaboration of the theory that underpins its experimental work. This initiative was repeated in 2023.

**LHCb student talks**

The LHCb student talks were originally a set of talks organized in 2008 with the intention of fostering collaboration between LHCb groups in the UK and funded by LHCb-UK. Since 2009, however this has evolved. The organization of these talks has been completed by student volunteers from UK institutes, with organizers changing every six months. The goal of LHCb student talks in recent years has been to have an expert explain a single aspect of the LHCb machine or method used in LHCb analyses. This has been found to be more useful for students compared to *e.g*. presentations of entire analyses. There has also been recent focus on “soft skills" such as thesis-writing and consideration of jobs post-Ph.D.

As these talks have grown, despite being funded by LHCb-UK, they are presented at CERN and regularly attended and given by people from any institute globally. The LHCb student talks are all archived and publicly available.^25^ Furthermore, since the global outbreak of COVID-19, there has been a renewed effort to record these talks, improving accessibility including those in other time zones.

### 2.4 Belle II

**Members: 1100+, Institutes: 300, Countries: 27**

The Belle II Collaboration (2010) is located at the SuperKEKB electron-positron collider in Tsukuba, Japan. It is designed for precision measurements of heavy quark and lepton physics involving *B* mesons and searches for new physics beyond the Standard Model. The Belle II collaboration currently consists of more than 1100 members from more than 300 institutions in 27 nations.^26^ A detailed summary of the current training model at Belle II can be found in Lieret et al. (2023).

#### 2.4.1 Primary induction to collaboration and core software training

**Belle II Software documentation**

Belle II employs a self-study friendly training model. Taking the disruption of in-person activities caused by the COVID-19 pandemic as an opportunity to rethink the previous training approaches, the Belle II software training material was remodeled to focus on self-study as the primary training mode. This reflects the observation that the efficiency and scope of synchronous training events are often not optimal, as the training needs, previous experiences, and schedule preferences diverge among the various new members. In addition, the reliance on slides and other hard-to-maintain material used in many in-person events results in high demand for personpower, duplicated efforts, and frequently outdated resources.

The framework for the new training material was therefore designed to specifically tackle the following challenges:

- A high degree of **maintainability and sustainability**. The most important aspect of this is **testability**: As far as possible, all examples should be tested against the current version of the Belle II software. This is important because the analyst-facing interface of the main software is still evolving. This also means that the training material should be versioned along with the main software.
- **Connecting resources**: In order to keep the material lean and avoid the duplication of content, the training material should be linked to relevant parts of the technical documentation (API documentation). Teaching newcomers to work with these resources should be an objective in itself.
- **Interactivity**: All lessons should be complemented by exercises with complete solutions and optional hints to adapt to the level of the learners.

To tackle these challenges, Belle II chose to directly host the documentation in the repository of the Belle II software framework (Moll, 2011) and render it via Sphinx^27^ together with the technical documentation. Every training module corresponds to one or more restructured text files. Importantly, Sphinx allows the inclusion of code from external source files. It also supports sophisticated ways to include *parts* of source files, which is important when building up larger examples step by step while avoiding duplicated code. Importing source code from external files allows running static code checkers and formatters on the code snippets and makes it easy to include them as unit tests. Unit testing ensures that no pull request that breaks backward compatibility can be merged without updating the relevant training material. To spot other issues even outside of events, anonymous feedback is solicited by integrating a small submission form at the bottom of every lesson.

The setup provides a comprehensive onboarding experience, covering everything from basic physics knowledge to software prerequisites and grid job submissions. Its effectiveness has been confirmed by several surveys of the participants of workshops shortly after the restructuring of the material. While some participants wished for additional interactive sessions and lectures, the material was very well received.

#### 2.4.2 Further software training beyond core curriculum

Whilst the integration of the training material with the Belle II software framework makes the setup more complex than that of a wiki or a stand-alone documentation system it offers advantages that justify the increased technical complexity. As discussed earlier, the setup ensures that all training examples remain functional through coupling with the software and performing unit tests. Furthermore, it allows for seamless linking between the tutorials and more technical documentation, pointing students to further material and in-depth resources without sacrificing clarity.

#### 2.4.3 Sources of continued training and support

One of the introductory modules describe the Belle II collaborative tools, including the available channels of communication for getting support if the participants have any questions related to the training materials. The Belle II documentation, including the training material, is publicly available^28^.

### 2.5 DUNE

**Members: 1600+, Institutes 207, Countries: 39**

The Deep Underground Neutrino Experiment (DUNE) is an international collaboration working to measure *CP* violation in neutrino oscillations and simultaneously observe supernova burst neutrinos. Additionally, the DUNE detectors will be sensitive to MeV scale solar neutrinos and will be used for rare and exotic BSM searches. DUNE consists of 1400 collaborators from over 200 institutions in over 30 countries. As of November 2024, there are 374 graduate students, 244 postdocs and 659 senior scientists with ~100 technical staff and interns. Descriptions of the science of DUNE are available for the public on the DUNE Science page^29^.

Members of the DUNE Computing Consortium have taken on the responsibility of providing documentation, basic computing tutorials, and technical support for computing users collaboration-wide. Members are committed to leveraging existing training materials provided by the open communities, and to contributing to the development of new learning modules with broadened appeal. We also use Fermilab docdb and CERN EDMS for archival documents.

#### 2.5.1 Primary induction to collaboration and core software training

**DUNE computing training**

While fundamentals such as using the Unix Shell, Git and GitHub, and Python are well covered by HSF and IRIS-HEP tutorials using the Software Carpentry (SC) lesson templates, DUNE-specific computing training (DeMuth et al., 2024) is provided as single and multi-day workshops that have generally been associated with collaboration meetings. Basic trainings have been offered 11 times; generally twice/year. Basics addressed are: how to access computing resources at Fermilab and CERN, data storage and management, event reconstruction and simulation using the art framework (Green et al., 2012) and LArSoft tools (Snider and Petrillo, 2017), and job submission and monitoring as a way to jump-start event simulation and reconstruction work. Typically 30-40 participants attend each tutorial event.

A small cadre of dedicated experts provide instruction utilizing SC lesson templates which are coded in Markdown, and who practice active learning techniques such as live-coding and quizzes. As GitHub hosted materials, GitHub-pages are used to render the materials as an elegant and interactive resource^30^.

A crucial part of the training, to ensure the ability of participants to actively follow the live exercises, is the pre-training setup that takes new users through the steps needed to ensure that they have valid computer accounts and can already access DUNE interactive computing resources at Fermilab or CERN. New users are invited to join a dedicated DUNE Slack^31^ channel to get assistance with setup and the tutorial itself. During training events, Google documents are used as “livedocs” for real-time responses to questions. Afterwards, livedocs become an archive resource that can be studied. This framework also operates well for asynchronous study with the captured Zoom videos of each session embedded. For those revisiting the lessons, and others who learn autonomously, asynchronous access to the lessons is encouraged. A recent survey of the collaboration indicated that the ability to do the tutorials independently was appreciated. Given the distributed nature of the collaboration and the costs of travel, we are moving toward offering more frequent, shorter tutorials over Zoom.

#### 2.5.2 Sources of continued training and support

DUNE has traditionally used a MediaWiki wiki^32^ to store documentation for computing and other projects. This wiki is password protected and suitable for information that should be restricted to the collaboration. DUNE is moving as much of its code and tutorials as possible to Github pages for open access.

Ensuring progress on the physics goals of DUNE requires user support in addition to documentation and training. Slack channels play an important role, particularly for asynchronous just-in-time support.

#### 2.5.3 Other learning resources

- A DUNE-specific glossary ^33^ is under development to help collaborators.
- DUNE Computing uses GitHub issues ^34^ as a mechanism for handling and archiving frequently asked questions.

## 3 Discussion

Table 1 summaries the main software training events offered by each collaboration. Along with the other induction events described in this paper they all have the same goal: to help new members of each experiment quickly learn the necessary skills to contribute to physics analysis and be efficient, well-integrated members of the collaboration. Some of the common themes and challenges when considering training and induction initiatives are discussed in this section. In Section 3.2, we summarize this discussion into a list of considerations for future initiatives.

**Table 1:** Summary of the main software training events offered by each collaboration.

**Collaboration**	**Members**	**Main training event**	**Format**
ATLAS	6000+	ATLAS analysis software tutorial	Separate in-person (CERN/US) and online events
CMS	6000+	CMS data analysis school	In-person typically at FermiLab or CERN
LHCb	1600+	StarterKit	Run in hybrid mode
Belle II	1100+	Belle II software documentation	Self-study focus
DUNE	1400+	DUNE computing training	Run in hybrid mode

### 3.1 Common themes and challenges

#### 3.1.1 Designing training events

With the advent of the COVID-19 pandemic, many training events shifted from in-person to remote, and currently many are offered in a hybrid format. Remote or hybrid events remove barriers to participation that travel can cause, and therefore allow more members of the collaboration to be trained. Similarly, the Belle II approach of self-study training materials ensures that all collaboration members can utilize the resources whenever they need to. One approach that several experiments have adopted is to record training events so that lectures can be watched by participants who were not able to attend the live events.

The primary benefit of in-person events is an improved ability to network and connect with other people from the same experiment. Often, data analysis schools or training events are the first opportunities that new members have to meet people outside of their own institution and to hear directly from experiment leadership. Knowing the internal structure of the collaboration is an important part of the induction process; LHCb and CMS have dedicated talks from the Spokesperson, Physics Coordinator, diversity officers and secretariats. When planning onboarding events, sufficient opportunities for networking with peers should be included, especially during hybrid or remote events. Grouping participants into teams to work on an example analysis (like what is done during the ATLAS and CMS tutorials) can also help facilitate meaningful networking as well as develop soft skills such as communication and teamwork skills. If possible, such in-person training and induction events should be scheduled directly before, after or even during collaboration weeks as practiced by DUNE. This can permit more early-career members to attend in person, since travel costs have to be paid only once.

Before live events, it is important to make sure that all participants have the necessary prerequisites to participate in the exercises. These prerequisites include computing accounts and certificates to use computing infrastructure, but also basic knowledge such as how to use Git or the Unix shell if these are not covered in the event itself. Pre-workshop checklists and dedicated support to fulfill said checklist should be setup to help participants prepare for synchronous events. Pre-workshop quizzes can also be used to assess the level of the incoming group and tailor content and timetabling. After the event, asking students for feedback can help improve future versions of the event. Having a clear way for participants to provide feedback for self-study training initiatives is also valuable as implemented at Belle II with anonymous feedback submission forms.

#### 3.1.2 Learning techniques

To increase retention of the material and student engagement, training initiatives should be interactive and incorporate active learning techniques. For example, in asynchronous training, this can be done with quizzes and exercises with solutions and hints. For synchronous events, live, hands-on exercises with direct support can complement traditional lectures. It is important to consider that there will be a range of abilities in any group and the rate at which people grasp new concepts will vary. Therefore extra help should be available where required and, whether synchronous or asynchronous, there should be a sufficient pool of instructors such that students can receive timely help if they are stuck on an exercise.

#### 3.1.3 Training materials

Training materials, even those designed for synchronous events, are often used as important reference materials year-round. Keeping these materials up-to-date is therefore essential to maximizing their impact. It should be expected that when one modifies experiment code, one also has to update the relevant documentation and tutorials. If the training materials are integrated into the experiment's software framework, like what is done in Belle II, then upkeep can be handled via continuous integration and unit tests. If there are regular synchronous events, then those are also natural opportunities to check all of the related documentation. The training materials should include links to other useful sources of (perhaps more technical) documentation within the experiment for further self-study. A glossary is a popular self-study resource designed to combat the wealth of jargon used within experiments that the whole collaboration should feel responsible for maintaining.

LHC experiments have also committed to the CERN Open Data policy (CERN, 2020) of making their data open for general consumption after a fixed period of time. The expected use cases of this Open Data are reinterpretation/reanalysis of physics results, education and outreach, technical and algorithmic development and physics research. Without good documentation on how to use the necessary (open source) experiment software, the CERN Open Data initiative will not fulfill its purpose. These onboarding software training materials should also then be recognized and used as resources toward the successful fulfillment of the CERN Open Data policy by the LHC experiments.

#### 3.1.4 Sustainability of training initiatives

Keeping training materials up-to-date and running training events takes a significant amount of personpower, and it is often a challenge to find enough people who have the time, ability, and motivation to make training initiatives successful. There are two complementary solutions to this problem: firstly, attracting new facilitators by emphasizing the benefits and rewards of assisting with training; and secondly, minimizing the required personpower by simplifying events or reducing the duplication of effort.

The first solution is to identify and motivate people to assist with training. An important part of this is making sure people's contributions to training are publicly acknowledged. The HSF Training Working Group (Malik et al., 2021), for example, maintains a central community website ^35^ listing everyone who has helped with one of their events. In CMS, many of the facilitators for the Data Analysis Schools or Hands-on Tutorial Programs come from the LPC Distinguished Researchers (DR) program. People selected as DRs gain visibility for their leadership roles within CMS, and there is an explicit expectation that DRs will assist with events at the LPC, including training events. Many experiments also follow the LHCb model, which has established a collaboration culture where participants are encouraged to become facilitators in a future round of the StarterKit. Belle II requires all software developers to contribute to training materials, since updating reference materials is an unavoidable step of the standard workflow. Experiments which have a ‘credit' system for service work could consider granting official credit for leading software training.

Researchers should also see the material as not just for new-starters but as an up-to-date reference guide for use throughout their involvement in the experiment. Many of the more mature collaboration members use the training material on a regular basis and should also be encouraged to contribute to its development and maintenance. This is particularly true for LHC experiments as we move into the HL-LHC era, when all collaboration members will have to become familiar with new software and tools. Finally, establishing the role of software engineer as a viable career path in HEP can help increase the pool of facilitators, especially if assisting with training is explicitly included in the job description.

The second solution is to run training initiatives as efficiently as possible, to reduce how many facilitators are needed. In part, this means avoiding duplication of effort and using existing resources as much as possible. Common skills such as proficiency with Python and C++ should be considered an asset across experiments, and tutorials on these topics should be run as such; LHCb has joined with ALICE and SHiP to deliver such lessons in the past. There are also regular events such as the PyHEP workshops and the HEP C++ Course^36^ with hands-on tutorials that, due to their wider nature and larger resource pool, can be more effective than events at collaboration level. The same applies to training materials; the HSF has developed the HSF Training Center^37^ with modules on cross-experiment topics which can be used to teach fundamental software and analysis skills. These are used effectively by some experiments and include, but are not limited to, Machine Learning, Git, Singularity/Docker, ROOT (Brun and Rademakers, 1997) and other programming languages.

The available personpower is also a key consideration when deciding on synchronous or asynchronous training. Asynchronous, self-study training materials such as those developed by Belle II, for example, require much less time than organizing extended live events, although in-person events have their own benefits as previously discussed. Another way to reduce development overheads is to make training material modular wherever possible. This helps people quickly access the particular task or “lesson” with which they need guidance and also aids in the maintenance of the material as it can be factorized out to different experts. Whole analysis examples (such as those used in the CMS and ATLAS tutorials) are valuable but also require significantly more time to develop and maintain.

#### 3.1.5 Long-lasting support

Throughout their career, when seeking support, collaboration members must ask well-formed questions in the appropriate communications channel. Asking good questions is a skill that can be taught to make interactions between newcomers and experts more efficient. This is recongnised in the LHCb Starterkit which has a dedicated lesson “Asking good questions”. Any training initiative should then also teach people (even senior members of the collaboration) about the support channels available and their remit as well as introduce participants to relevant experts or peers on particular topics.

By providing these skills participants should feel empowered to ask questions and seek help when they run into issues applying the training to real-world analysis tasks. There is a wide variety of methods for providing real-world support, including Git issues, Slack or Mattermost channels. It is observed that for more informal channels like Slack and Mattermost there is a lower perceived barrier for newcomers to ask questions.

### 3.2 Summary and considerations for future experiments

All large experiments should have comprehensive induction and training schemes not only for efficient onboarding of new members but also collaboration cohesion. To summarize the discussion we propose a list of recommendations for training and induction schemes at large experiments:

**Induction and training events**

- Events should be held at least once a year, preferably before or after other collaboration events to help with travel costs. Whilst it would be unreasonable to make these events mandatory they should be highly encouraged by the experiments' management
- Provide pre-workshop checklists (eg. computing accounts, certificates) with dedicated hands-on support sessions to help students complete the checklist before the event
- To improve accessibility, have the option of online participation for those who are not able to attend in-person events along with dedicated online-only instructors
- Include an introduction to collaboration structure and introductory talks from relevant offices where possible
- Provide networking opportunities for new members of the collaboration, for instance, through group activities. This is also possible for online participants through the use of “breakout rooms"
- Implement metrics for assessing the quality of events such as anonymous post-event feedback surveys. This can be used to inform changes to the material and its delivery.
- Follow proven pedagogical practices such as active learning techniques like quizzes to engage participants and increase retention of the material
- Ensure hands-on sessions have a good ratio of instructors to students with dedicated online instructors for hybrid events
- Reward and motivate the hard work done by facilitators so that training is viewed as a rewarding and vital task within the collaboration

**Induction and training materials**

- Keep material modular where possible. Whilst whole analysis examples are valuable, modular training material aids maintenance and findability
- Keep training materials up-to-date all year round so they can serve as a useful reference for self-study increasing accessibility. Additionally have a support channel for those choosing the self-study option
- Take advantage of, and contribute to, the existing training resources such as the many experiment-agnostic training modules provided by the HSF
- Encourage senior members of the collaboration to contribute to the upkeep of software training material, since they can also benefit from it as a valuable reference
- In the training material introduce participants to channels for future support and provide advice on how to ask good questions eg. how to create minimal reproducible examples
- Metrics such as the number of views for a given tutorial (outside of dedicated events) can provide information on their relevancy or otherwise and indicate where effort should be directed

Finally, leaders of experiment training events should continue to communicate with their colleagues in other experiments so common lessons, challenges, and solutions can be shared. Improved training initiatives across HEP contributes positively to the physics goals of each experiment, which is beneficial for our entire field.

## 4 Conclusions

This paper documents and reviews the approaches taken to onboarding new members of the ATLAS, CMS, LHCb, Belle II and DUNE collaborations. By documenting and sharing these initiatives experiments can be aware of each others' practices and a collective knowledge can be built. Through analyzing the experiences of these experiments, the HSF has summarized a set of recommendations for onboarding new members into scientific collaborations. Most importantly, scientific collaborations should incentivize the enormous effort, and recognize the importance of, delivering and maintaining training events/materials. To minimize duplicated efforts, where possible, resources should be shared within the community, and materials provided by groups such as the HSF should be exploited. For future experiments, which will see even greater data volumes and larger collaborations these training and onboarding considerations are crucial for ensuring maximal physics output.

Future work in this area involves a comparative analysis of the effectiveness of different approaches, gathering common metrics across the experiments. With this we can look to identify optimal methods of delivery of training material. For instance, one could compare the project-based learning in CMS against the self-study tutorials of Belle II aiding future design. An example of such an immediate metric is post-training participant surveys (as some experiments are already performing); these can be complemented by surveying members reaching their first full year in the collaboration to assess, on reflection, how well the training prepared them for their work on the experiment.

## Footnotes

^1^HEP Software Foundation (https://hepsoftwarefoundation.org/).

^2^DIANA-HEP: Data-Intensive Analysis for High Energy Physics (https://diana-hep.org/).

^3^IRIS-HEP: Institute for Research and Innovation in Software for High Energy Physics (https://iris-hep.org/).

^4^HSF Training Hackathon (https://indico.cern.ch/event/975487/).

^5^Data analysis training in HEP experiments (https://indico.cern.ch/event/1175097/ and https://indico.cern.ch/event/1175096/).

^6^The ATLAS Collaboration (https://atlas.cern/Discover/Collaboration).

^7^ATLAS Early Career Scientist Board (https://atlas.cern/authors/atlas-early-career-scientists-board).

^8^How to do ATLAS analysis from start to finish: a hands on tutorial (https://indico.cern.ch/event/1181243/).

^9^ATLAS Software Documentation (https://atlassoftwaredocs.web.cern.ch/).

^10^ATLAS Analysis Tutorial Week (https://atlassoftwaredocs.web.cern.ch/ASWTutorial/TutorialWeek/).

^11^ATLAS Experiment on YouTube (https://www.youtube.com/@ATLASExperiment/videos).

^12^CMS People Statistics (https://cms.cern/collaboration/people-statistics).

^13^LPC Data Analysis School (https://lpc.fnal.gov/programs/schools-workshops/cmsdas.shtml).

^14^ https://mattermost.com/

^15^LPC Distinguished Researcher program (https://lpc.fnal.gov/fellows).

^16^LPC Hands-On Tutorial Sessions (https://lpc.fnal.gov/programs/schools-workshops/hats.shtml).

^17^ https://www.zoom.com

^18^HSF Analysis Essentials (https://hsf-training.github.io/analysis-essentials/).

^19^Starterkit First Analysis Steps (https://lhcb.github.io/starterkit-lessons/first-analysis-steps/README.html).

^20^HSF PyHEP working group (https://hepsoftwarefoundation.org/workinggroups/pyhep.html).

^21^The Gauss Project (http://lhcbdoc.web.cern.ch/lhcbdoc/gauss/).

^22^Moore Documentation (https://lhcbdoc.web.cern.ch/lhcbdoc/moore/master/index.html).

^23^Starterkit Second Analysis Steps (https://lhcb.github.io/starterkit-lessons/second-analysis-steps/README.html).

^24^Implications of LHCb measurements and future prospects workshop, October 2023 (https://indico.cern.ch/event/1166230/).

^25^LHCb student talks archive (https://twiki.cern.ch/twiki/bin/view/Main/LHCbUKStudentGroupMeetingArchive).

^26^Belle II Collaboration (https://belle2.jp/the-collaboration/).

^27^ https://www.sphinx-doc.org/

^28^Belle II Analysis Software Framework (basf2) (https://github.com/belle2/basf2).

^29^DUNE Science public page (https://www.dunescience.org/).

^30^DUNE Computing Training, 2024 edition (https://dune.github.io/computing-basics/).

^31^ https://slack.com/

^32^https://www.mediawiki.org/wiki/MediaWiki/, https://wiki.dunescience.org/

^33^ABC Dune (https://abc.dunescience.org/).

^34^DUNE FAQ (https://github.com/orgs/DUNE/projects/19).

^35^HSF Training Community (https://hepsoftwarefoundation.org/training/community.html).

^36^HEP C++ Course (https://github.com/hsf-training/cpluspluscourse).

^37^HSF Software Training Center (https://hepsoftwarefoundation.org/training/curriculum.html).
